# Editorial: Exploring the diversity, ecological significance, and systematics of uncultivated prokaryotic taxa

**DOI:** 10.3389/fmicb.2025.1604849

**Published:** 2025-05-08

**Authors:** Maher Gtari, Louis S. Tisa, Marike Palmer, Jean Armengaud

**Affiliations:** ^1^Department of Biological and Chemical Engineering USCR Molecular Bacteriology and Genomics (BMG), National Institute of Applied Sciences and Technology, University of Carthage, Tunis, Tunisia; ^2^Department of Molecular, Cellular, and Biomedical Sciences, University of New Hampshire, Durham, NC, United States; ^3^Department of Microbiology, University of Manitoba, Winnipeg, MB, Canada; ^4^Department of Medications and Technologies for Health (DMTS), University of Paris-Saclay, CEA, INRAE, SPI, Bagnols-sur-Cèze, France

**Keywords:** uncultivated, bacteria, archaea, omics, SeqCode, *Candidatus*, taxogenomics

The vast majority of prokaryotes have not yet been cultivated due to several factors, including low abundance, slow growth rates, unknown growth requirements, dependency or interactions with other organisms (Gutleben et al., 2018; Overmann et al., 2017; Vartoukian et al., 2010; Lewis et al., 2021). Additionally, the immense biodiversity and unexplored habitats in which these organisms exist, along with their potential disinclination for cultivation using conventional methods, have made the study of these uncultivated prokaryotes a critical frontier in microbiology, unveiling extensive and previously unexplored microbial diversity (Rinke et al., 2013; Huber et al., 2007; Parks et al., 2017; Lloyd et al., 2018). Despite significant challenges, recent advancements in omics technologies have provided invaluable insights into the ecological roles, systematics, and metabolic capacities of these elusive microorganisms (Garza and Dutilh, 2015; Lewis et al., 2021). Traditionally, many prokaryotes have been considered difficult or even impossible to culture using conventional methods, discouraging many microbiologists from studying them and leaving much of the microbial diversity undiscovered. However, innovative approaches leveraging metagenome-assembled genomes (MAGs) and single-cell amplified genomes (SAGs) are now shedding light on their biology, potentially guiding the development of tailored cultivation strategies and challenging the long-held paradigm of “uncultivability.” These techniques significantly broaden our understanding of microbial diversity, ecological interactions and roles, and evolutionary history (Gutleben et al., 2018; Lewis and Ettema, 2019; Lewis et al., 2021; Xie et al., 2021; Liu et al., 2022; Laugier, 2023). Coupled with innovative culturing techniques such as co-cultivation strategies, microfluidics, and synthetic biology, these developments are establishing culturomics as a valuable complement to omics-based studies. Further pushing the boundaries of microbial discovery is essential for unraveling the functioning of complex microbial communities that have long been unknown or have evaded traditional cultivation methods (Lagier et al., 2012; Nowrotek et al., 2019).

The seven studies presented in this Research Topic highlight the dynamic roles of uncultivated prokaryotes across diverse ecological settings, progress in their genomic characterization, and implications for systematics. These papers illustrate how advances in omics technologies are deepening our understanding of microbial diversity, ecological significance, and taxonomy. They emphasize the growing importance of genomic data in unraveling the complex ecological functions of uncultivated taxa and their potential contributions to improve applications in biotechnology and environmental science.

- Shah et al. investigate prokaryotic communities in deep-sea regions rich in polymetallic nodules. Using next-generation sequencing, their study maps microbial diversity and reveals how these microbes contribute to biogeochemical cycles in the deep ocean, enhancing our understanding of marine ecology.
- Rodríguez-Cruz et al. examine microbial diversity in the unique Cuatro Ciénegas Basin. Through MAGs, they explore the genomes of bacterial and archaeal communities from this extreme ecosystem, providing insights into microbial adaptation to harsh conditions and illustrating the value of genomic data in ecological studies.
- Gtari et al. address challenges in cultivating *Frankia* and *Protofrankia* species, key nitrogen-fixing microsymbionts of actinorhizal plants. Their comparative genomic analysis suggests strategies for overcoming cultivation barriers by examining metabolic pathways and ecological traits that may guide the development of more effective cultivation media and conditions.
- Cao et al. reveal the genomic and physiological properties of *Anoxybacterium hadale* gen. nov. sp. nov., a novel anaerobic bacterium isolated from the Mariana Trench's hadal zone. This discovery expands our knowledge of microbial life in extreme environments and highlights the importance of dissolved organic sulfur in microbial metabolism. The study also introduces a new member to the class *Clostridia* and offers valuable insights into the biogeochemical cycles of deep-sea ecosystems.
- Miklós et al. explore the role of environmental bacteria in the population dynamics of *Hydra* species under various temperature conditions. Their findings demonstrate how bacteria, previously considered mere environmental factors, significantly influence the growth and survival of organisms in cooler environments, affecting population growth, and ecosystem stability.
- Pittino et al. investigate the ecology of Tintenstrich communities, focusing on microbial communities on rock surfaces in Switzerland. Their analysis shows that Cyanobacteria and Proteobacteria dominate these environments, with distinct microbial diversity patterns between siliceous and carbonate substrates. This study enhances our understanding of lithic microbial communities and their ecological functions, such as nutrient release into water systems.
- Ho et al. introduce a novel genus and species, *Candidatus* Pelagadaptatus aseana, derived from marine metagenomes from Singapore's protected coastal waters. This work underscores the power of MAGs in discovering previously unculturable microbes. The proposed genus, representing a distinct lineage with unique metabolic pathways, highlights the role of uncultivated bacteria in nutrient cycling and ecological functions, such as nitrogen reduction in seawater. Additionally, the reclassification of *Umboniibacter* to the newly established family *Umboniibacteraceae* fam. nov. reflects ongoing advances in prokaryotic taxonomy driven by genomic insights.

These studies collectively demonstrate the growing significance of omics in advancing our understanding of microbial diversity, ecological significance, and systematics of uncultivated taxa. As research continues to explore these uncharted microbial realms, it is becoming increasingly clear that the uncultivated prokaryotic world holds the keys too many crucial ecological processes and evolutionary mysteries. This collection of research not only enhances our knowledge of microbial systems but also opens a wealth of new possibilities for biotechnology and environmental management.

In conclusion, exploring uncultivated prokaryotes offers a vital perspective for understanding life's complexities across diverse environments. As these studies demonstrate, the future of microbiology lies not only in studying cultivated microbes but also in deciphering the genomes, functioning, and ecological roles of those yet to be cultured.
